# Continuous Resonance Tuning without Blindness by Applying Nonlinear Properties of PIN Diodes

**DOI:** 10.3390/s21082816

**Published:** 2021-04-16

**Authors:** Yong Luo, Hongtao Liu, Yiming He, Hengrong Cui, Guangli Yang

**Affiliations:** 1 School of Communication and Information Engineering, Shanghai University, Shanghai 200444, China; y_luo@foxmail.com (Y.L.); LHT_shu@163.com (H.L.); yiming_he@foxmail.com (Y.H.); guangli.yang@shu.edu.cn (G.Y.); 2 College of Information Sciences and Technology, Donghua University, Shanghai 201620, China

**Keywords:** active metamaterial antenna, continuous tuning, resonance blindness, EM co-simulation, nonlinear property

## Abstract

Metamaterial antennas consisting of periodical units are suitable for achieving tunable properties by employing active elements to each unit. However, for compact metamaterials with a very limited number of periodical units, resonance blindness exists. In this paper, we introduce a method to achieve continuous tuning without resonance blindness by exploring hence, taking advantage of nonlinear properties of PIN diodes. First, we obtain the equivalent impedance of the PIN diode through measurements, then fit these nonlinear curves with mathematical expressions. Afterwards, we build the PIN diode model with these mathematical equations, making it compatible with implementing co-simulation between the passive electromagnetic model and the active element of PIN diodes and, particularly, the nonlinear effects can be considered. Next, we design a compact two-unit metamaterial antenna as an example to illustrate the electromagnetic co-simulation. Finally, we implement the experiments with a micro-control unit to validate this method. In addition, the nonlinear stability and the supplying voltage tolerance of nonlinear states for both two kinds of PIN diodes are investigated as well. This method of obtaining smooth tuning with nonlinear properties of PIN diodes can be applied to other active devices, if only PIN diodes are utilized.

## 1. Introduction

**Figure 1:**
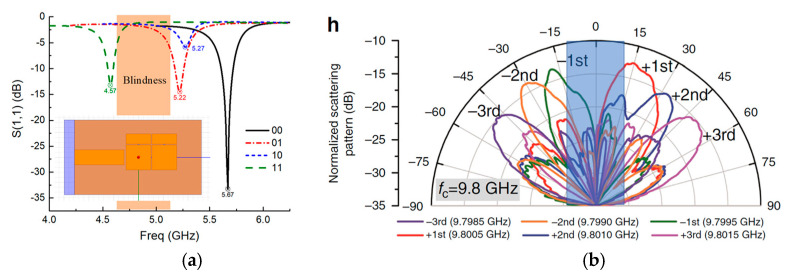
Tuning blindness exists in (**a**) a compact MTM antenna with two periodical units and in (**b**) programmable spatial radiation patterns [9].

Electromagnetic metamaterials (EM MTMs) [1] employ periodical units, that are derived from split-ring resonators (SRRs) [2], composite right–left-handed (CRLH) structures [3], and high-impedance structures (HISs) [4], to obtain a negative refractive index, negative phase constant, and high surface impedance, thereby achieving the unique properties of super-lens [5], back–forward radiation [6], and field enhancement [7]. Thanks to EM MTMs that are characterized by periodical configuration, it is possible to realize multiple tunable states either in spectrum resonances [8] or spatial radiation patterns [9] by applying active components to each periodical unit. This kind of tuning mechanism benefits from a periodical array with n unit cells, while each unit can be tuned individually with m states utilizing active elements such as PIN diodes [8,10,11,12], varactors [13,14,15,16,17,18,19,20], or MEMS [21], thus, ideally speaking, we can possess in total as many as m^n^ tunable states. This means that extremely large MTMs with an infinite (*n*→∞) number of units have an infinite number of tunable states, leading to continuous tuning. Conversely, compact MTMs with a very limited number of units have only several discrete tunable states. The absence of continuous tunability in an active MTM design is called tuning blindness, and it is has two causes: the MTM design has very limited periodical units, such as two, three, or five cells; RF switches as the active component in the MTM only have two tunable states (ON/OFF). For instance, as demonstrated in Figure 1a, we simulate an MTM antenna containing two-unit (*n* = 2, *m* = 2) HIS structures, and it indeed demonstrates several tunable resonances, but they are discrete with unavoidably induced resonance blindness (as shown in the shadow area). Similarly, in [9], where programmable radiations are realized with PIN diodes, and in [21], where programmable spectrum resonances are achieved with MEMS, there exists tuning blindness as well. More specifically, in [9], though scanning beams from roughly −60° to +60° are obtained, as the shadow area demonstrates in Figure 1b, scanning blindness occurs from −15° to +15°. In this paper, we explore another method to achieve continuous tuning with PIN diodes: investigating the equivalent impedance in the transition zone between completed ON and OFF, and exploring the nonlinear zone in between. Thanks to the PIN diodes possessing this nonlinear zone, we can achieve a continuous spectrum tuning without blindness and, meanwhile, with low actuated voltages less than 1.5 V, which is suitable for NB-IoT scenery that requires many tunable but narrow-band spectrum channels and with low power consumption.

In practice, MTMs with a limited number of periodical units are quite common, and in some cases, they are even preferred due to their compact size. In addition to the method proposed in this paper, another common method for avoiding blindness is to increase the tunable states *m* possessed by the individual cell, while unit number *n* is kept to a small value for a compact size. For instance, [13,14,15,16,17,18,19,20] introduce the tunable antenna using varactor diodes, or variable capacitors, to obtain multiple tunable states *m* = 9, 7, 6, respectively. These good works with multi-state tuning indeed increase the tuning continuity with discrete structures, but usually require variable voltage to even as high as 20 V, which might not be compatible with low-power applications such as narrow bandwidth Internet of Things (NB-IoT) [22,23,24]. 

This paper is arranged as follows: Section 2 investigates the nonlinear property of PIN diodes; Section 3 introduces the electromagnetic (EM) co-simulation; Section 4 presents experiments; Section 5 and Section 6 provide the discussion and conclusion.

## 2. Nonlinear Properties

**Figure 2:**
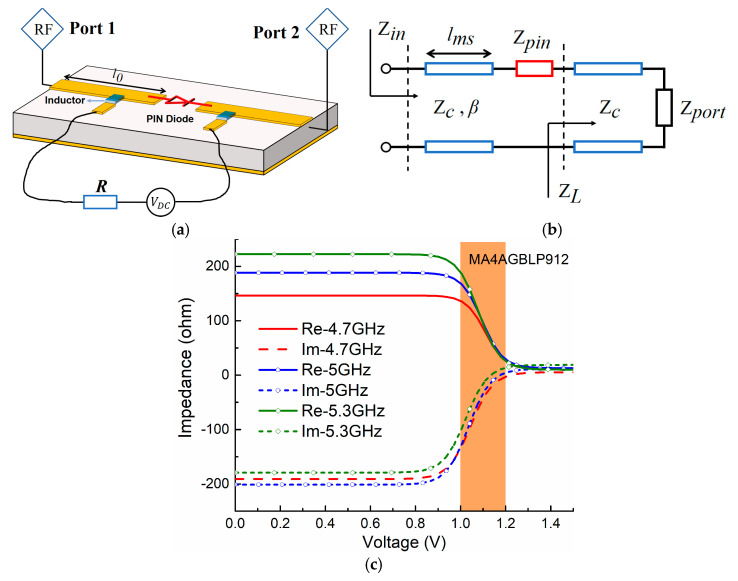
(**a**) Measurement setup and (**b**) its equivalent circuit model for investigating the nonlinear zone of PIN diode A (MACOM MA4AGBLP912); (**c**) extracted equivalent impedance including resistance and reactance according to different voltages actuated to the PIN diode.

PIN diodes are conventionally utilized as RF switches with two states (ON/OFF). However, there exists a transition zone in between. In order to investigate this nonlinear property, we study the relationship between the equivalent impedance and the actuated voltage using the PIN diode A (MACOM MA4AGBLP912, MACOM, Lowell, MA, USA). First, we measure the PIN diode by employing a microstrip line in a 5 GHz band. As shown in Figure 2a, we make a slot in the middle of the standard 50 Ω microstrip line and integrate the surface-mounted PIN diode A there, then apply two inductors (Murata LQW18AN22NG00, Mutrata, Nagaokakyo, Kyoto, Japan) with a large value (22 nH) to block the interference from the DC supplier. Second, we apply transmission line (TL) theory to analyze this equivalent circuit model, as shown in Figure 2b. The equivalent model includes *Z_c_* = 50 Ω that represents the characteristic impedance of the standard transmission line with length *l*_0_, the equivalent impedance *Z_pin_* of the PIN diodes, and the port impedance *Z_port_* = 50 Ω. According to TL theory, the equivalent impedance of PIN diodes *Z_pin_* can be retrieved from input impedance *Z_in_* as [25]

(1) $$Z_{\mathrm{pin}}=\frac{Z_{C}{\cdot (Z}_{\mathrm{in}}-Z_{L}{)+j\cdot \tan(\beta l}_{0}{)\cdot (Z}_{\mathrm{in}}{\cdot Z}_{L}-Z_{C}^{2})}{Z_{C}-{j\cdot Z}_{\mathrm{in}}{\cdot \tan(\beta l}_{0})}$$

where *β* is the phase constant, and the input impedance *Z_in_* is measured in experiments.

As shown in Figure 2c, since actuated voltages are varied from 0 V to 1.5 V, the equivalent impedance of the PIN diodes *Z_pin_* is changed accordingly; the resistance ranges from 225 Ω to a very small value close to 0 Ω, and the reactance varies from −200 Ω to a very small value as well. Particularly, we can observe that there exists a transition zone (as marked by the shadow area in Figure 2c) between the PIN diodes’ OFF zone where impedance is around 200–200 j, and the ON zone where the impedance is a very small value close to zero. In this transition zone, the actuated voltage is around 1–1.2 V and, accordingly, the impedance varies nonlinearly and smoothly from the OFF state to the ON state.

In order to accommodate the EM co-simulation including passive EM models and nonlinear active components, we build a PIN diode model with respect to the nonlinear properties and considering parameters of actuated voltages and frequencies. Referring to impedance curves as shown in Figure 2c, curves in the transition zone are nonlinear in an S shape, which is close to the Boltzmann function [26]. Thus, we select the Boltzmann function to fit them. Based on Boltzmann’s mathematical model, the real part *Z_Re_* and imaginary part *Z_Im_* are

(2) ZRe=ZRe_on+ZRe_off - ZRe_on1+eV-VRe_0dRe

(3) ZIm=ZIm_on+ZIm_off - ZIm_on1+eV-VIm_0dIm

in which *V* is the actuated voltage for PIN diode A, *Z_Re_off_* and *Z_Re_on_* are the measured *Z_Re_* when the diode is in the OFF state with *V* = 0 V and the ON state with *V* = 1.5 V. Similarly, *Z_Im_off_* and *Z_Im_on_* are *Z_Im_* when *V* = 0 V (OFF state) and 1.5 V (ON state). *V*_*Re*_0_ is defined as the voltage when *Z_Re_* equals the mean of *Z_Re_off_* and *Z_Re_on_*, while *V*_*Im*_0_ is the voltage when *Z_Im_* equals the mean of *Z_Im_off_* and *Z_Im_on_*. Parameters *d_Re_* and *d_Im_* are the slope of curves *Z_Re_* and *Z_Im_* when *V* = *V*_*Re*_0_ and *V* = *V*_*Im*_0_. 

Until now, the above equations have concerned only one frequency point, but we need to consider the whole frequency band. This means all the parameters in (2) and (3), *Z_Re_off_*, *Z_Re_on_*, *V*_*Re*_0_, *d_Re_* and *Z_Im_off_*, *Z_Im_on_*, *V*_*Im*_0_, *d_Im_*, need to be related to frequencies. We select several frequency points located at the relatively low, moderate, and high frequency sections of the band, and fit them to the equations, thereby involving the whole frequency band when describing the nonlinear properties. Particularly, according to these curves’ shapes concerning frequencies, the mean function and Gaussian function are applied to fit the real part *Z_Re_* and the imaginary part *Z_Im_*, respectively. For *Z_Re_*, the relative parameters in respect to frequencies can be described as

(4) ZRe_on=13∑f=f0, f1, f2ZRe(f)

(5) ZRe_off=ZRe_off(f2)+d1⋅(f−f2)/109

(6) VRe_0=VRe_0(f2)+d2⋅(f−f2)/109

(7) dRe=dRe(f2)+d3⋅(f-f2)/109

where three typical frequency points are *f*_0_ = 4.7 GHz, *f*_1_ = 5 GHz, and *f*_2_ = 5.3 GHz. Other parameters are *Z_Re_on_* = 11.18 Ω, *Z_Re_off_* (*f*_2_) = 223.8 Ω, *V*_*Re*_0_ (*f*_2_) = 1.08 V, *d_Re_* (*f*_2_) = 0.04751, *d*_1_ = 126.57, *d*_2_ = −0.05024, and *d*_3_ = 7.45 × 10^−3^. Similarly, we use (8)–(11) for the imaginary part *Z_Im_*:

(8) ZIm_on=ZIm_on(f2)+d4⋅(f−f2)/109

(9) ZIm_off=ZIm_off(f2)+d5⋅e-0.5⋅(f/109−f3d6)2

(10) VIm_0=VIm_0(f2)+d7⋅(f−f2)/109

(11) dIm=dIm(f2)+d8⋅(f−f2)/109

where the relative parameters *Z_Im_on_* (*f*_2_) = 18.63 Ω, *Z_Im_off_* (*f*_2_) = −171.38 Ω, *V*_*Im*_0_ (*f*_2_) = 1.023 V, and *d_Im_* (*f*_2_) = 0.04902. Other parameters *d*_4_ = 22.59, *d*_5_ = −27.24, *d*_6_ = 0.23727, *d*_7_ = −0.03697, and *d*_8_ = 7.966·10^−4^. Especially, the parameter *f*_3_ = 4.922 is derived from the peak position of the Gaussian function. Finally, we achieve the completed equations to express the nonlinear property of the PIN diode as follows

(12) ZRe=13∑f=f0, f1, f2ZRe(f)+ZRe_off(f2)+d1⋅(f-f2)/109−13 ∑f=f0,f1,f2ZRe(f)1+eV−(VRe_0(f2)+d2⋅(f−f2)/109)dRe(f2)+d3⋅(f-f2)/109

(13) ZIm=ZIm_on(f2)+d4⋅(f-f2)/109+ZIm_off(f2)+d5⋅e−0.5⋅(f/109−f3d6)2− (ZIm_on(f2)+d4⋅(f−f2)/109)1+eV−(VIm_0(f2)+d7⋅(f−f2)/109)dIm(f2)+d8⋅(f-f2)/109

**Figure 3:**
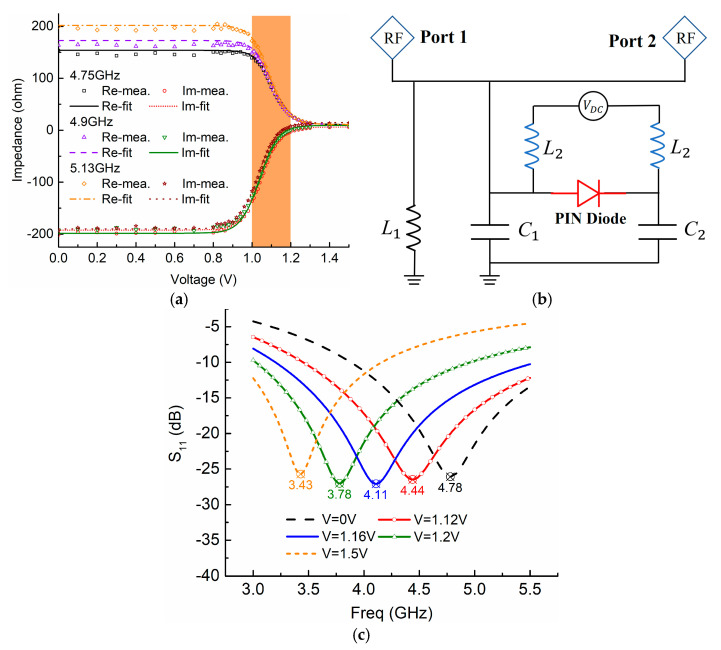
(**a**) Comparisons between fitting results by using the Boltzmann function and measured results; (**b**) the circuit contains parallel LC and PIN diode A (MACOM MA4AGBLP912) model, thereby implementing the proof-of-concept simulation to prove (**c**) continuous and smooth resonance tuning without blindness.

Note that we fit the measured impedance curves of the PIN diode with these abovementioned equations through several typical frequency points *f*_0_, *f*_1_, and *f*_2_, thus, we need to double check if they can represent the whole frequency band. We randomly select the frequencies 4.75 GHz, 4.9 GHz, and 5.13 GHz in the band, and compare the fitting curves with the measured results. As shown in Figure 3a, *Z_Re_* and *Z_Im_* match well with the measured ones, implying the equivalent effectiveness of the nonlinear property in the whole frequency band. In this way, we obtain the mathematical expressions to describe the nonlinear properties of the PIN diode, and accordingly model this PIN diode in ANSYS Electronics Desktop, ensuring the nonlinear property is considered in the EM co-simulation.

To demonstrate that the nonlinear properties can be exploited for achieving smooth and uniform resonance tuning, we implement a proof-of-concept level simulation with the PIN diode A (MACOM MA4AGBLP912). As shown in Figure 3b, it is a parallel *L*_1_*C*_1_ circuit model with the parameters *L*_1_ = 1 nH and *C*_1_ = 1 pF. IN particular, we put another capacitance *C*_2_ in the shunt direction with the same value *C*_2_ = 1 pF, but it can be connected or disconnected parallel to the *L*_1_*C*_1_ circuit via the PIN diode, which can be controlled by the supplying voltages from the OFF state, nonlinear states to ON state. An inductance of *L*_2_ = 1 H is used to block the interference from the DC suppliers. Theoretically speaking, there should be a continuous resonance tuning between the resonance $1/\left(2\pi \sqrt{L_{1}C_{1}}\right)=5.03$ GHz when the PIN diode is ideally open, and $1/\left(2\pi \sqrt{{2L}_{1}C_{1}}\right)=$ 3.56 GHz when the diode is ideally short, through middle states while actuating the diode in the nonlinear zone. As shown in Figure 3c, with controlling the actuated voltages to make the PIN diode work in OFF, ON, and transition states, the resonances are tuned from 3.43 GHz to 4.78 GHz via nonlinear states 3.78 GHz, 4.11 GHz, and 4.44 GHz, respectively. This continuous and smooth resonance tuning verifies the concept of eliminating the resonance blindness with nonlinear properties by PIN diodes.

In a brief summary, PIN diodes have the advantages of nonlinear properties while the actuated voltages fall in the transition zone, providing the potential capability of continuous tuning in MTM antenna even with a very limited number of units. That is either different from varactors that rely on a large dynamic voltage range, or different from MEMS that have a noncontinuous equivalent capacitance value variation due to the beam membrane pulled in the 1/3 length position [27].

## 3. Layout, Design, and EM Co-Simulation

**Figure 4:**
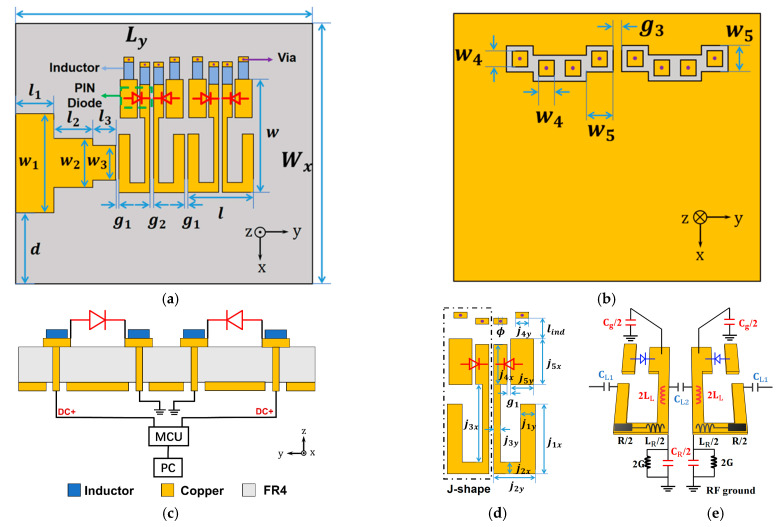
Design model of the proposed MTM antenna: (**a**) top view, (**b**) bottom view, (**c**) side view, and (**d**) unit design with dimensions (mm): W_x_ = 26, L_y_ = 34.5, l_1_ = 3.5, w_1_ = 9, l_2_ = 6.5, w_2_ = 4, l_3_ = 2, w_3_ = 2.7, w = 9.1, l = 5.2, g_1_ = 0.2, g_2_ = 0.3, d = 7.85, j_1x_ = 4.7, j_1y_ = 0.9, j_2x_ = 0.8, j_2y_ = 2.5, j_3x_ = 5.2, j_3y_ = 0.4, j_4x_ = 2.7, j_4y_ = 0.8, j_5x_ = 3.1, j_5y_ = 1.4, g_4_ = 0.4, l_ind_ = 1.4, Φ = 0.2, w_4_ = 0.8, w_5_ = 1.4; (**e**) the equivalent CRLH circuit model of unit cell.

We design a compact MTM antenna using PIN diodes to introduce the EM co-simulation, and take advantage of its nonlinear properties to realize the smooth tuning and eliminate the resonance blindness. As in Figure 4a, the active MTM structure comprises two periodical cells which produce a compact size, a PIN diode that plays the role of the active element in each unit, and inductance chips for blocking the interference from DC suppliers. Via holes are made between the top and bottom layers (Figure 4b) to connect the micro-control unit (MCU) for DC supply. As seen in the side view in Figure 4c,d, PIN diodes placed in the two slots of each unit electrically connect/disconnect these slots, thus manipulating zeroth-order resonances (ZORs) of the MTM antenna. Thanks to the MTM configuration separating units from each other, voltages for actuating each PIN diode can be controlled independently. FR4 material with permittivity *ε_r_* = 4.3, tan*δ* = 0.02, and thickness *h* = 2.5 mm is used as the substrate. The unit cell is designed according to CRLH-TL theory [11], in which the equivalent circuit model has inductances and capacitances in both series and shunt directions, thereby producing the zeroth-order resonance (ZOR) resonating at the frequency *β* = 0. The mechanism can be qualitatively demonstrated by the equivalent circuit model, as shown in Figure 4e. The left-handed capacitance is equivalently considered as *C_L_* = 2*C*_*L*1_ + *C_L_*, where capacitance *C*_*L*1_ is formed by the gaps between adjacent units and *C*_*L*2_ is produced by the two symmetric J-shaped patches. Left-handed inductance *L_L_* is generated by a strip patch in the *x*-direction, and is regarded as being connected to the ground through another capacitance *C_g_* induced between the edge patch and the ground. Series inductance *L_R_* and shunt capacitance *C_R_* are formed from the conventional microstrip line. According to CRLH theory, the ZOR *ω_zor_* is related to shunt-directed resonances *ω_sh_* [11]:

(14) $$\omega_{zor}=\omega_{sh}=\sqrt{\frac{1}{L_{L}C_{g}}+\frac{1}{L_{L}C_{R}}}$$

which implies what the active element PIN diodes are particularly utilized to tune: shorting/opening PIN diodes alter the effective area of the edge patch, leading to equivalently varying the capacitance *C_g_*, hence, tuning the ZORs. Moreover, we design the unit operating in the ZOR mode, because at this resonance, the phase constant *β* = 0, and its guided wavelength, is infinite, leading to the favorable characteristic that its resonance is independent of the physical length [11]. Therefore, we have the freedom to employ arbitrary numbers of periodical units. For a compact MTM antenna to demonstrate ZOR tuning without blindness, we utilize two periodical units as an example.

**Figure 5:**
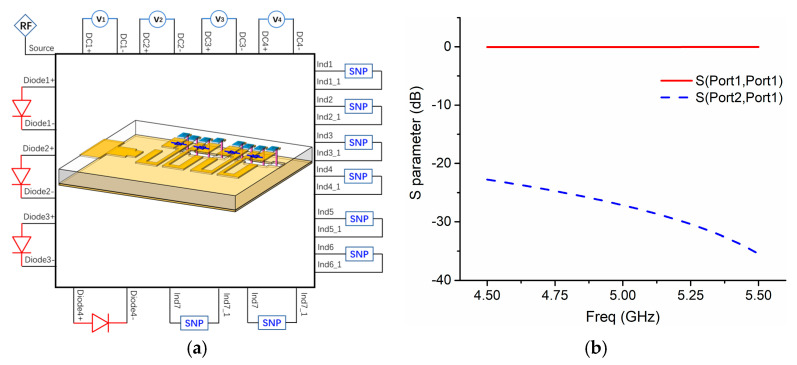
(**a**) EM co-simulation model including passive EM model, active element PIN diodes, and inductance chip; (**b**) simulated S-parameters from the SNP file of inductor (Murata LQW18AN22NG00)**.**

We use ANSYS Electronics Desktop to simulate the whole design including the passive EM model and the active element PIN diodes, as illustrated in Figure 5a. First, we design the antenna with passive simulation in HFSS without any diodes. That means in the passive full-wave simulation, with/without rectangular patches are utilized to imitate ON/OFF states of PIN diodes, thus considering a preliminary simulation with electric field distributions and radiation patterns. Afterward, in the EM model, lumped ports are set up where the active elements are placed, so we have chances to insert the active element model there. Then, we build the SPICE model for PIN diode A MACOM MA4AGBLP912 with the mathematical equations shown above, thereby involving its nonlinear property. In addition, the S2P file of the inductor (Murata LQW18AN22NG00, Mutrata, Nagaokakyo, Kyoto, Japan) is employed in the EM co-simulation as well. As shown in Figure 5b, a simulation is conducted with the S2P file of the inductor, and it exhibits good isolation of less than −20 dB between the DC supplier and RF signals. As the active elements are ready, finally, we can implement the EM co-simulation by considering the S2P file of the inductor and the SPICE model of PIN diode A for the lumped ports. Particularly, four DC voltage sources are connected to the lumped ports as well to supply PIN diodes accordingly. In such a method, we can achieve the results of co-simulation easily within a few minutes.

**Figure 6:**
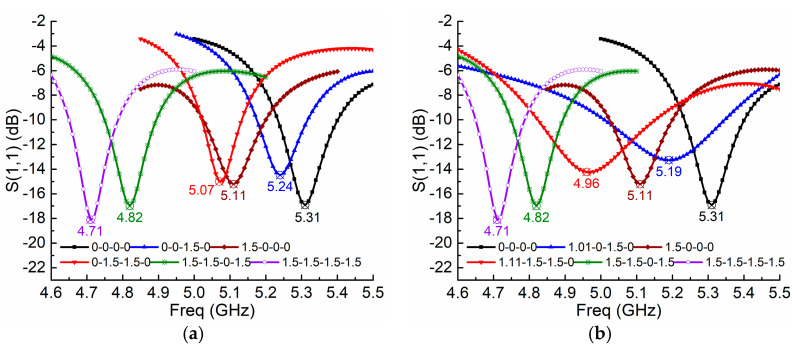

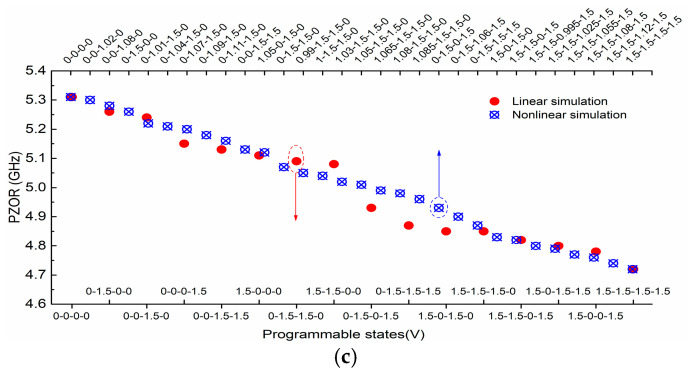
Simulation results of S11 with co-simulation method using PIN diode model as RF switch: (**a**) 6 linear states; (**b**) 6 nonlinear states with uniform tuning; and (**c**) ZOR comparisons of all 16 linear states with 30 nonlinear states.

With the co-simulation method, we obtain the active MTM antenna simulations as plotted in Figure 6, in which both the linear and nonlinear cases are illustrated. In the linear case, as shown in Figure 6a, there are OFF and ON states, and we code PIN diodes in the OFF state as state 0 when 0V is applied, and code the ON state as state 1.5 when 1.5 V is applied. For example, 0-1.5-0-1.5 means the second and fourth PIN diodes are actuated to the ON state while other two diodes are in the OFF state. In a nonlinear case, as shown in Figure 6b, however, more nonlinear states where actuated voltages fall in the transition zone are shown. We code these nonlinear states exactly as the voltages actuated to PIN diodes. For instance, 0-1.1-1.23-1.09 indicates the four PIN diodes are actuated with 0 V, 1.1 V, 1.23 V, and 1.09 V, respectively. In Figure 6b, the ZORs of the nonlinear case are tuned from 4.71 GHz to 5.31 GHz via 4.82 GHz, 4.96 GHz, 5.11 GHz, and 5.19 GHz, while in the linear case, as shown in Figure 6a, the resonances are tuned from 4.71 GHz to 5.31 GHz but the tuning is not smooth and continuous, and there is blindness in the band from 4.83 GHz to 5.07 GHz. Since each unit can provide four coding sequences, 0-0, 0-1.5, 1.5-0, and 1.5-1.5, an MTM antenna consisting of two units has all 16 coding states to cover 4.71 GHz to 5.31 GHz, while for the nonlinear case, it has more middle states. As shown in Figure 6c, by comparing the 16 states of the linear case and 30 selected states of the nonlinear case, we find that nonlinear advantages allow the ZOR tuning to be smooth, continuous, and uniform, without resonance blindness.

In summary, we apply the S2P file of the inductor, the SPICE model of PIN diode A, and the DC voltage model to the EM co-simulation. These kinds of two-port models have the advantages of not needing to consider the complicated equivalent circuit model with all detailed parameters of R, L, and C, because all of these circuit parameters are included in the S2P model or SPICE model. Thanks to the EM co-simulation considering nonlinear properties of PIN diodes, we can simulate an active MTM antenna with continuous and uniform resonance tuning, and eliminate the resonance blindness. The nonlinearity of PIN diodes not only prevents frequency tuning blindness due to the compact MTM design with limited discrete states, but also makes frequency tuning uniform.

## 4. Experimental Implementation

**Figure 7:**
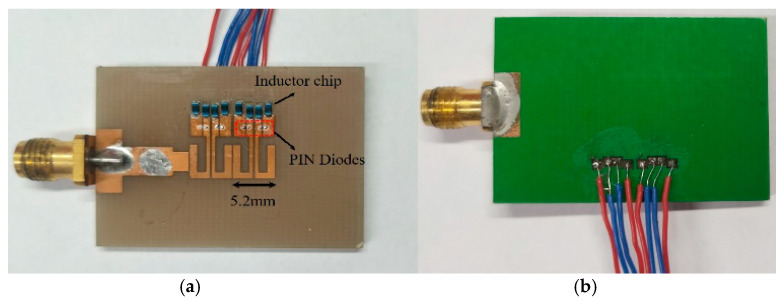

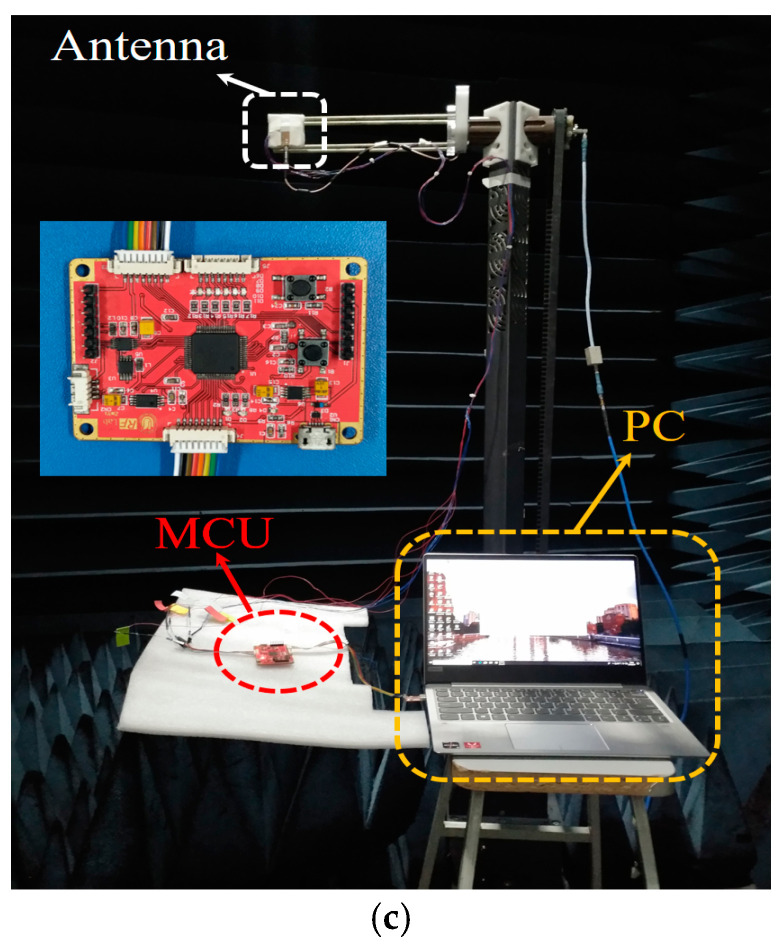
Fabricated MTM antenna includes (**a**) top view of MTM units with PIN diodes and inductor chips; (**b**) bottom view of four pairs of wires for DC voltage supply; (**c**) anechoic chamber measurements in which a PC controls the MCU for outputting DC voltages to PIN diodes.

According to the previous design, the compact MTM antenna consisting of two cells is fabricated as shown in Figure 7a. The configuration and layout are exactly that in Figure 4: the FR4 substrate has the parameters of *ε_r_* = 4.3, tan*δ* = 0.02, and PIN diode A and the inductance chip are MACOM MA4AGBLP912 and Murata LQW18AN22NG00, respectively. As demonstrated in Figure 7b, via holes go through the substrate to connect four pairs of wires, so as to supply these PIN diodes through the micro-control unit (MCU). In this design, four channels of the DC supply can be manipulated independently because of the isolated and periodical configuration of the MTM. Figure 7c shows the setup for anechoic chamber measurements, in which a laptop is utilized to output C language for controlling the MCU for voltage manipulations.

**Figure 8:**
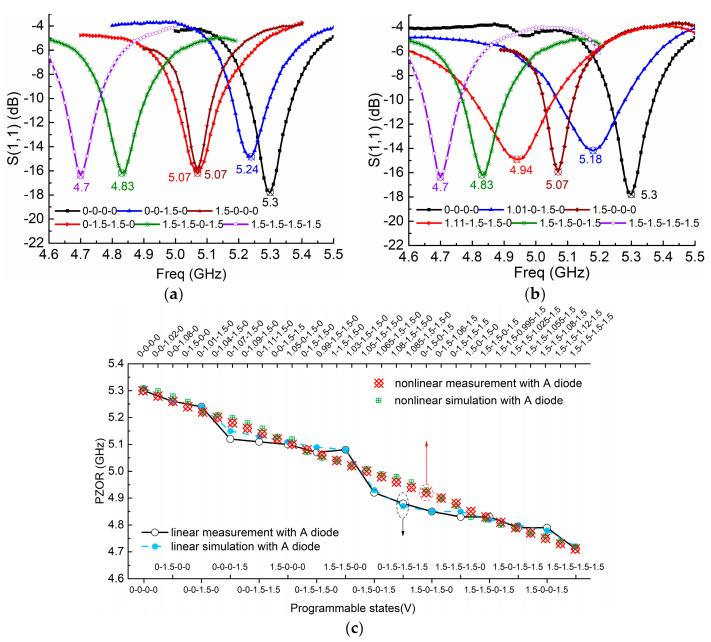
(**a**) Measured ZOR tuning in linear case including states: 0-0-0-0, 0-0-1.5-0, 1.5-0-0-0, 0-1.5-1.5-0, 1.5-1.5-0-1.5, 1.5-1.5-1.5-1.5 and (**b**) in nonlinear case including states: 0-0-0-0, 1.01-0-1.5-0, 1.5-0-0-0, 1.11-1.5-1.5-0, 1.5-1.5-0-1.5, and 1.5-1.5-1.5-1.5; (**c**) ZOR comparisons between linear case of all 16 linear states, and nonlinear case of 30 states.

We measure both the linear case, which includes OFF (actuated voltage 0 V, indicated as 0) and ON states (actuated voltage 1.5 V, indicated as 1.5), and the nonlinear case (state coded as the actuated voltage) which considers applying voltages in the transition zone. In Figure 8a,b, several ZORs of the linear and nonlinear cases are demonstrated, and it is seen that as resonances are tuned from 4.7 GHz to 5.3 GHz via many tuning states, the resonance tuning of the linear case is not uniform, while that of the nonlinear case is uniform and smooth. More specifically, as shown in Figure 8c, more tunable states are compared. For the linear case, which considers all the completed *m^n^* = 4^2^ = 16 tuning states (*m* = 4 represents the four PIN diodes, and *n* = 2 indicates PIN diodes’ ON/OFF states) for the compact two-cell MTM antenna, we can clearly observe that its tuning is nonuniform and blindness clearly exists in the frequency band of 4.9~5.1 GHz and 5.1~5.25 GHz. For instance, states 0-0-1.5-1.5 and 1.5-0-0-0 have almost the same resonant point and overlap at 5.12 GHz, while states 1.5-1.5-0-0 and 0-1.5-0-1.5 are separated by roughly 0.2 GHz and are recognized as tuning blindness. For the nonlinear case with manipulated supply voltages in the transition zone of 1 V to 1.2 V, however, the tuning is very uniform, leading to continuous resonance tuning without blindness. In this case, we code the supplied voltage of the PIN diode working in the nonlinear zone. For example, state 1.05-1.5-1.5-0 means the four PIN diodes from left to right are actuated with 1.05 V, 1.5 V, 1.5 V, and 0 V, respectively. Thanks to the PIN diode possessing the nonlinear property, we can obtain many tunable states. Thirty tunable states are illustrated in Figure 8c, and it is seen that ZORs are tuned uniformly with a step around 0.02 GHz in the range from 4.7 GHz to 5.3 GHz, eliminating the resonance blindness and indicating the nonlinear advantages of PIN diodes. In addition, as shown in Figure 8c, in both linear and nonlinear cases, simulated ZORs agree well with measured ones, validating the effectiveness of the nonlinear model and EM co-simulation.

Note that, as shown in Figure 8b, the bandwidth is varied when resonances are tuned in different states. This can be explained by the fact that when the active element PIN diodes are used to tune the effective area of the edge patch, they vary circuit parameter *C_g_*, as shown in Figure 4e. Meanwhile, the PIN diode itself induces resistance as well, which varies the conductance *G*. Hence, the *Q* factor and bandwidth are changed. More specifically, according to the CRLH theory, the resonance *ω_zor_* is dominated by the shunt-directed resonances *ω_sh_*, as indicated in Equation (14). Thus, the *Q* factor and bandwidth are investigated and discussed in terms of the shunt-directed circuit part. As shown in Figure 4e, which illustrates the equivalent circuit model, the shunt admittance can be written as

(15) $$Y=j\omega C_{R}-\frac{j\omega C_{g}}{\omega^{2}L_{L}C_{g}-1}+G$$

The quality factor *Q* is

(16) $$Q=\frac{1}{2}\frac{\omega }{G}\left(\frac{C_{R}^{2}+\omega^{2}C_{R}^{2}LC_{g}+C_{R}C_{g}}{C_{g}}\right)$$

Consequently, the bandwidth can be expressed as

(17) $$BW=\frac{\omega }{2\pi Q}=\frac{GC_{g}}{2\pi C_{R}\left(C_{g}+C_{R}\right)}$$

This equation can qualitatively explain the relationship between the bandwidth and different tunable states. Employing PIN diodes in an active MTM antenna electrically opens/shorts the gaps in the edge patch, resulting in varying the parameter *C_g_*. On the other hand, the resistance variations in the PIN diodes in the shunt direction affect the conductance *G*. That indicates that tunable states vary both the *C_g_* and *G*. According to Equation (17), these two variables change the bandwidth. Therefore, as seen in Figure 8b, the bandwidth is changed according to different tunable states.

**Figure 9:**
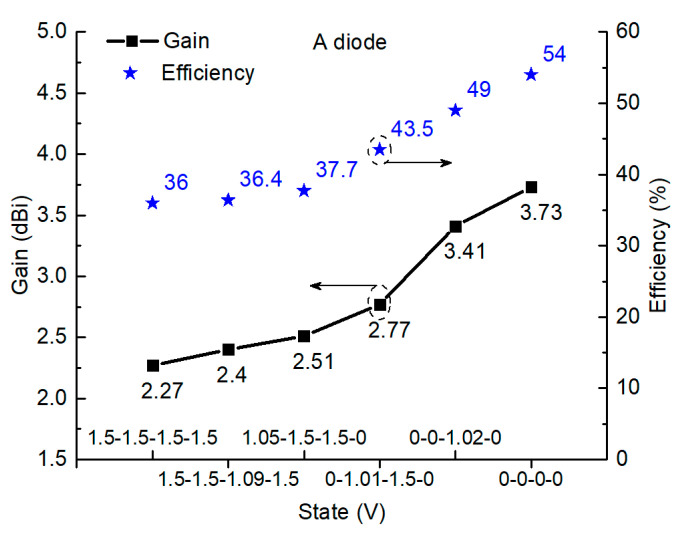
Measured gain and efficiency of the active antenna in two extreme states and four nonlinear states with PIN diode A (MACOM MA4AGBLP912).

**Figure 10:**
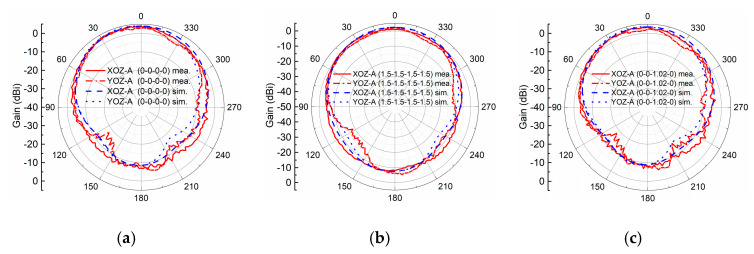

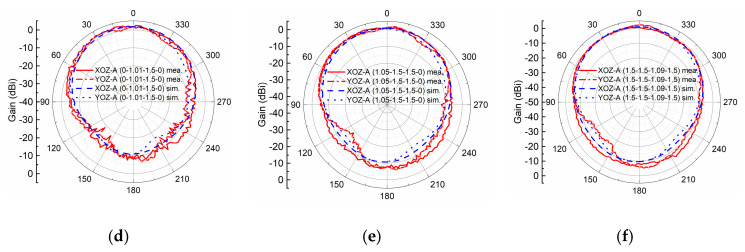
Comparisons between simulated and measured radiation patterns of the two extreme states: (**a**) 0-0-0-0 and (**b**) 1.5-1.5-1.5-15, and four nonlinear states: (**c**) 0-0-1.02-0, (**d**) 0-1.01-1.5-0, (**e**) 1.05-1.5-1.5-0, and (**f**) 1.5-1.5-1.09-1.5.

We study the gains, efficiency, and radiation patterns of the active MTM antenna with PIN diode A (MACOM MA4AGBLP912). In particular, two extreme states of completed ON and OFF states and four nonlinear states are investigated, while in other states, gains and the radiation efficiency are on the same level, and radiation patterns are quite similar. As shown in Figure 9, two extreme states, 0-0-0-0 and 1.5-1.5-1.5-1.5, that indicate PIN diodes are completely OFF/ON, have gains of 3.73 dBi and 2.27 dBi, respectively. For another four nonlinear states, 0-0-1.02-0, 0-1.01-1.5-0, 1.05-1.5-1.5-0, and 1.5-1.5-1.09-1.5, the measured gains are 3.41 dBi, 2.77 dBi, 2.51 dBi, and 2.4 dBi, which are between the gains of the two extreme cases. The corresponding radiation efficiencies of the nonlinear states are 49%, 43.5%, 37.7%, and 36.4%, which are between the two extreme states of 54% (OFF state) and 36% (ON state). In terms of radiation patterns, as illustrated in Figure 10a–f, all the states, including completed ON/OFF states and four nonlinear states, demonstrate similar radiation patterns, and the measured radiation patterns agree well with the simulated patterns.

In this part, based on the EM co-simulation, we implement experiments with PIN diodes, which demonstrate nonlinear advantages over the linear case in eliminating resonance blindness, and in realizing uniform and continuous ZOR tuning. 

## 5. Discussion

**Figure 11:**
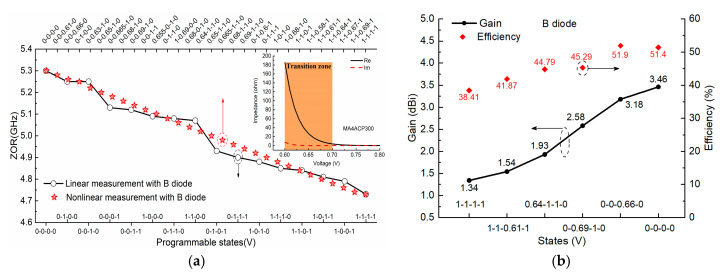
(**a**) Similar nonlinear advantages over linear case in achieving uniform tuning with diode B working in the transition zone; (**b**) measured gains and radiation efficiency of the two extreme states and four nonlinear states.

In this section, several interesting items associated with the nonlinearity of PIN diodes are discussed. First, we keep the same MTM antenna design but change it to employ PIN diode B (MACOM MA4FCP300), to study the generality of this kind of nonlinear property. As shown in Figure 11a, it demonstrates similar nonlinear properties: there exists a nonlinear zone where the actuated voltages fall in the transition zone 0.6–0.7 V, and by taking advantage of the nonlinear property, we can achieve similar nonlinear advantages over the linear case in achieving uniform and continuous ZOR tuning without blindness in the range 4.7 GHz to 5.3 GHz. Relative radiation patterns are quite similar to that of PIN diode A, and gains and the radiation efficiency are illustrated in Figure 11b; gains and radiation efficiency for the two extreme cases are 1.34 dBi and 3.46 dBi, and 38.41% (ON state) and 51.4% (OFF state), respectively, while for other nonlinear states of 0-0-0.66-0, 0-0.69-1-0, 0.64-1-1-0, and 1-1-0.61-1, the relative values are on the same level but between that of the completed ON and OFF states. That means, whether for PIN diode A or B, the nonlinear property is not a special case and can exist similarly and generally in other kinds of PIN diodes.

**Figure 12:**
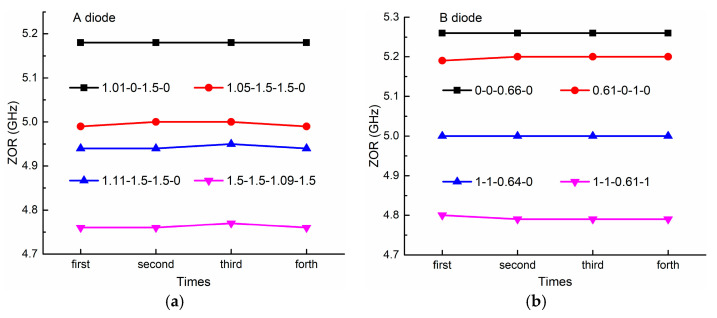
The stability of the nonlinear states with (**a**) PIN diode A and (**b**) PIN diode B.

Second, we study the stability of the nonlinear property, namely, how stable the PIN diodes are while they work in the nonlinear zone. As shown in Figure 12a, we measure the four nonlinear states of 1.01-0-1.5-0, 1.05-1.5-1.5-0, 1.11-1.5-1.5-0, and 1.5-1.5-1.09-1.5 when using PIN diode A (MACOM MA4AGBLP912) and another four nonlinear states of 0-0-0.66-0, 0.61-0-1-0, 1-1-0.64-0, and 1-1-0.61-1 when using PIN diode B (MACOM MA4FCP300, MACOM, Lowell, MA, USA), four times on different dates. In the measurements for the two different PIN diodes, the ZORs are kept the same with a slight variation, indicating good stability of the nonlinear property. For example, for PIN diode A, state 1.01-0-1.5-0 provides the same resonance at 5.18 GHz at different measurement times, and other nonlinear states have variations less than 0.005 GH.

**Figure 13:**
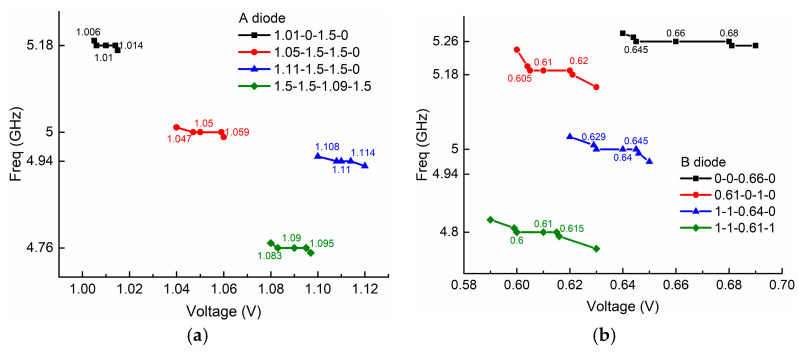
The supplying voltage tolerance of nonlinear states of (**a**) PIN diode A (**b**) PIN diode B.

Third, voltage tolerance needs to be investigated because ZORs seem very sensitive to voltage variation when PIN diodes operate in the nonlinear transition zone. Figure 13a shows the supplying voltage has good tolerance and gets rid of the risk of excessive sensitive voltage variations regardless of the type of PIN diode. For instance, considering state 1.01-0-1.5-0 for PIN diode A, we can achieve a stable resonant frequency at 5.18 GHz despite varying the supplying voltage from 1.005 V to 1.015 V, meaning that we have a voltage tolerance of 0.01V. In terms of PIN diode B with the state 0.61-0-1-0, as shown in Figure 13b, similarly, we achieve a stable resonant frequency of 5.18 GHz despite varying the supplying voltage from 0.605 V to 0.621 V, which indicates that we have a voltage tolerance of 0.016 V.

**Figure 14:**
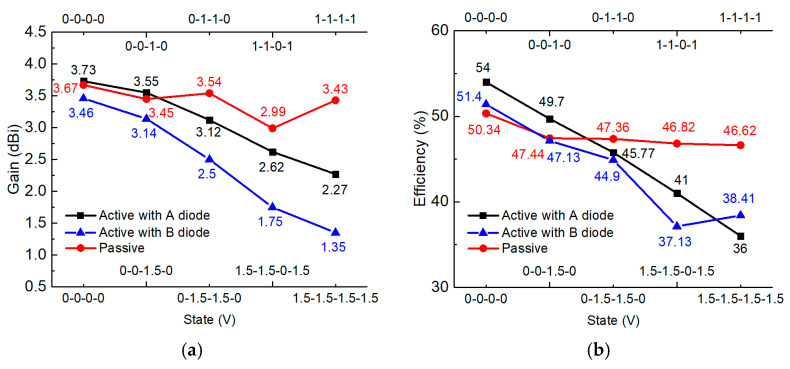
Comparisons between the active antenna and passive antenna with both PIN diodes A and B for (**a**) measured gains and (**b**) the measured efficiency.

Finally, for the proposed active MTM antenna, we investigate the influence of the active components, including inductance, MCU, and PIN diodes on radiation gains and the efficiency. Looking at Figure 14a, several states for both active and passive cases are shown, and it is seen that the gains with active components decrease by 1 or 2 dBi compared to those without active components, while the radiation efficiency of the active case, as shown in Figure 14b, is lower than that of the passive case but no more than 10%. 

Employing active components, as compared in Table 1, indeed shows the nonlinear advantages in eliminating resonance blindness over the passive case or the case only applying the RF switches with only OFF/ON states. Meanwhile, this proposed active MTM antenna requires actuated voltages lower than 1.5 V, which can be applied to 5G narrow bandwidth Internet of Things (NB-IoT) with low power capacities.

## 6. Conclusions

In this paper, we study the nonlinear property of PIN diodes, fit it to an EM co-simulation, and, particularly, apply it to an active MTM antenna to eliminate resonance tuning blindness. We conclude that the nonlinear property indeed possesses the advantages to help achieve smooth resonance tuning with low actuated voltages, and it can be generally extended to other PIN diodes with good stability and voltage tolerance. The active MTM antenna with uniform and smooth frequency tuning slices the frequency spectrum into many narrow-band channels, which can be applied to 5G narrow bandwidth Internet of Things (NB-IoT), which requires spectrum channels of narrow bandwidth and low power capacities.

## Figures and Tables

**Table 1:** Comparison of different types of tuning.

References	Active Devices	Number	Tuning Style	Tuning Stated	Bias Voltage (V)	Resonance Blindness
[8]	PIN diode	15	discrete	1.97 GHz and 2.37 GHz	0–0.7	yes
[10]	PIN diode and varactor diode	2 and 2	continuous	3.04 GHz to 5.89 GHz	0–30	no
[12]	PIN diode	6	discrete	5.95 GHz and 7.2 GHz	/	yes
[14]	Varactor diode	1	continuous	1.6 GHz to 2.23 GHz	2–20	no
[17]	Varactor diode	2	continuous	1.94 GHz to 2.44 GHz	0–20	no
This work	PIN diode	4	continuous	4.7 GHz to 5.3 GHz	0–1.5	no

## Data Availability

The data presented in this study are openly available.

## References

[1] Veselago V.G. (1968). The electro dynamics of substances with simultaneously negative values of ε and μ. Sov. Phys. Usp..

[2] Smith D.R., Padilla W.J., Vier D.C., Nemat-Nasser S.C., Schultz S. (2000). Composite Medium with Simultaneously Negative Permeability and Permittivity. Phys. Rev. Lett..

[3] Lai A., Caloz C., Itoh T. (2004). Composite right/left-handed transmission line metamaterials. IEEE Microw. Mag..

[4] Sievenpiper D., Zhang L., Broas R., Alexopolous N., Yablonovitch E. (1999). High-impedance electromagnetic surfaces with a forbidden frequency band. IEEE Trans. Microw. Theory Tech..

[5] Giovampaola C.D., Engheta N. (2014). Digital metamaterials. Nat. Mater..

[6] Sievenpiper D. (2005). Forward and backward leaky wave radiation with large effective aperture from an electronically tunable textured surface. IEEE Trans. Antennas Propag..

[7] Cai J., Zhou Y., Zhang Y., Li Q. (2018). Gain-assisted ultra-high-Q spoof plasmonic resonator for the sensing of polar liquids. Opt. Express.

[8] Khan M.S., Capobianco A.-D., Iftikhar A., Asif S., Ijaz B., Braaten B.D. (2016). A Frequency-Reconfigurable Series-Fed Microstrip Patch Array with Interconnecting CRLH Transmission Lines. IEEE Antennas Wirel. Propag. Lett..

[9] Zhang L., Chen X., Liu S., Zhang Q., Zhao J., Dai J., Bai G., Wan X., Cheng Q., Castaldi G. (2018). Space-time-coding digital metasurfaces. Nature Commun..

[10] Li T., Zhai H., Li L., Liang C. (2014). Frequency-Reconfigurable Bow-Tie Antenna with a Wide Tuning Range. IEEE Antennas Wirel. Propag. Lett..

[11] Caloz C., Itoh T. (2005). Electromagnetic Metamaterials: Transmission Line Theory and Microwave Applications.

[12] Qin P.-Y., Weily A.R., Guo Y.J., Bird T.S., Liang C.-H. (2010). Frequency Reconfigurable Quasi-Yagi Folded Dipole Antenna. IEEE Trans. Antennas Propag..

[13] Kim J., Kim G., Seong W., Choi J. (2009). A Tunable Internal Antenna with an Epsilon Negative Zeroth Order Resonator for DVB-H Service. IEEE Trans. Antennas Propag..

[14] Mirzaei H., Eleftheriades G.V. (2011). A Compact Frequency-Reconfigurable Metamaterial-Inspired Antenna. IEEE Antennas Wirel. Propag. Lett..

[15] Boukarkar A., Lin X.Q., Jiang Y. (2015). A Dual-Band Frequency-Tunable Magnetic Dipole Antenna for WiMAX/WLAN Applications. IEEE Antennas Wirel. Propag. Lett..

[16] Yu Y., Xiong J., Li H., He S. (2011). An Electrically Small Frequency Reconfigurable Antenna with a Wide Tuning Range. IEEE Antennas Wirel. Propag. Lett..

[17] Huang H.-J., Tsai C.-H., Lai C.-P., Chen S.-Y. (2016). Frequency-Tunable Miniaturized Strip Loop Antenna Fed by a Coplanar Strip. IEEE Antennas Wirel. Propag. Lett..

[18] Chi P.-L., Waterhouse R., Itoh T. (2011). Compact and Tunable Slot-Loop Antenna. IEEE Trans. Antennas Propag..

[19] Ko J., Kim D. (2017). A Wideband Frequency-Tunable Dipole Antenna Based on Antiresonance Characteristics. IEEE Antenna Wirel. Propag. Lett..

[20] Takemura N. (2012). Tunable Inverted-L Antenna with Split-Ring Resonator Structure for Mobile Phones. IEEE Trans. Antennas Propag..

[21] Chaabane G., Madrangeas V., Chatras M., Arnaud E., Huitema L., Blondy P. (2017). High Linearity 3-Bit Frequency Tunable Planar Inverted F-Antenna for RF Applications. IEEE Antennas Wirel. Propag. Lett..

[22] Narayanan S., Tsolkas D., Passas N., Merakos L. NB-IoT: A Candidate Technology for Massive IoT in the 5G Era. Proceedings of the 2018 IEEE 23rd International Workshop on Computer Aided Modeling and Design of Communication Links and Networks (CAMAD).

[23] Mahjoubi A.E., Mazri T., Hmina N. NB-IoT and eMTC: Engineering Results Towards 5G/IoT Mobile Technologies. Proceedings of the 2018 International Symposium on Advanced Electrical and Communication Technologies (ISAECT).

[24] Yang W., Wang M., Zhang J., Zou J., Hua M., Xia T., You X. (2017). Narrowband Wireless Access for Low-Power Massive Internet of Things: A Bandwidth Perspective. IEEE Wirel. Commun..

[25] Pozar D.M. (2007). Transmission Line Theory. Microwave Engineering.

[26] Dubois J.-M., Ouanounou G., Rouzaire-Dubois B. (2009). The Boltzmann equation in molecular biology. Prog. Biophys. Mol. Biol..

[27] Luo Y., Xu J., Yang G., Toshiyoshi H. (2018). EM radiation from electrostatic nonlinear pull-in instability of MEMS. Electron. Lett..

